# LEA proteins and ABA signaling: reciprocal regulation in stress adaptation

**DOI:** 10.3389/fpls.2025.1715223

**Published:** 2025-12-11

**Authors:** Cuihong Hao, Xinxin Zhan, Ning Guo, Jing Liu, Dayong Cui

**Affiliations:** 1 Shandong Engineering Research Center of Rose Breeding Technology and Germplasm Innovation, School of Life Sciences, Qilu Normal University, Jinan, China; 2 Key Laboratory of Biological Resources and Ecology of Pamirs Plateauin Xinjiang Uygur Autonomous Region, College of Life and Geographic Sciences, Kashi University, Kashi, China

**Keywords:** LEA proteins, abiotic stress, abscisic acid, stress resistance mechanism, regulatory network

## Abstract

Challenging environmental conditions are major factors that severely affect plant growth and limit agricultural productivity. To mitigate these stresses, plants have evolved various adaptive mechanisms. Among these, Late Embryogenesis Abundant (LEA) proteins play a pivotal role in responding to abiotic stresses and participate in a reciprocal regulatory network with the abscisic acid (ABA) signaling pathway. However, the precise molecular mechanisms underlying this reciprocity and the full composition of this network require systematic integration. This review synthesizes recent advances to propose a novel “ABA-LEA feedback loop” model and presents a comprehensive analysis of the classification into seven groups, structural features, molecular functions and mechanisms by which LEA proteins contribute to plant stress resistance. Special emphasis is placed on the intricate interplay between LEA proteins and the ABA signaling pathway, encompassing both the ABA-dependent regulation of *LEA* expression and the reciprocal feedback exerted by LEA proteins on ABA signaling through mechanisms that influence ABA homeostasis and signaling. By synthesizing evidence for this reciprocal regulation, this review establishes a novel feedback loop model that redefines LEA proteins as active modulators rather than passive effectors in stress signaling, offering new theoretical targets for breeding stress-resilient crops.

## 1 Introduction

Abiotic stresses, such as drought, heat, cold and excess salt, result in significant challenges to plant growth and productivity. In response, plants activate complex adaptive mechanisms that include hormonal signaling, transcriptional reprogramming, and the activation of protective proteins (Waadt et al., 2022; Zhang et al., 2022). Among these responses, LEA proteins play a pivotal role as molecular protectors. Initially identified for their seed-specific accumulation during cotton embryogenesis (Dure et al., 1981; Bojórquez-Velázquez et al., 2019), LEA proteins are now recognized as key stress resistance factors, ubiquitously expressed across plant organs (roots, stems, leaves) and in phylogenetically diverse organisms (Battaglia et al., 2008; Du et al., 2013; Charfeddine et al., 2015; Liu et al., 2019a; Knox-Brown et al., 2020; Kosová et al., 2021; Hsiao, 2024). Their distinct biophysical properties (exceptional thermostability, high hydrophilicity, and resistance to denaturation) facilitate the stabilization of cellular structures under extreme environmental conditions (Guo et al., 2023).

*LEA* expression is primarily regulated by ABA, a central signaling molecule that coordinates stress-response networks (Leprince et al., 2017; Müller et al., 2017). Emerging evidence indicates a reciprocal relationship between LEA proteins and ABA pathways, where LEA proteins both respond to and actively modulate ABA signaling, suggesting bidirectional crosstalk within a more extensive stress-adaptation network. Despite comprehensive genomic characterization of LEA families across diverse taxa (Battaglia et al., 2008; Liu et al., 2019a; Knox-Brown et al., 2020; Kosová et al., 2021), significant knowledge gaps remain regarding their underlying functional mechanisms: the evolutionary divergence of LEA structural and functional traits across different plant lineages remains insufficiently explored; mechanistic insights into LEA-mediated stress protection remain fragmented across various studies; and the regulation between LEA proteins and ABA signaling has yet to be systematically integrated.

In this review, we synthesize existing research by systematizing the classification and structural principles of LEA proteins, elucidating their mechanistic roles in abiotic stress mitigation, and proposing a unified model for dynamic LEA-ABA signaling interactions. This analysis aims to guide future engineering of stress-resistant crops through targeted manipulation of LEA-based regulatory networks.

## 2 Structural characteristics and classification of LEA proteins

LEA proteins, which are recognized for their critical roles in plant stress tolerance, constitute a family of hydrophilic polypeptides (Szlachtowska and Rurek, 2023). These proteins typically possess conserved sequence motifs, characterized by repeated arrangements of hydrophilic residues, including glycine (Gly), alanine (Ala), and glutamate (Glu) (Hundertmark and Hincha, 2008; Du et al., 2013). Despite this sequence conservation, LEA proteins exhibit structural plasticity. Computational and experimental studies reveal that they generally lack stable secondary structures in solution, classifying them as intrinsically disordered proteins (IDPs) (Hincha and Thalhammer, 2012; Wang et al., 2024b). Remarkably, their conformation is stress-responsive: under hydration they remain disordered, whereas under dehydration they reversibly fold into ordered α-helices (Hundertmark et al., 2011; Rendón-Luna et al., 2024). This structural transition is fully reversible upon rehydration (Hincha and Thalhammer, 2012).

This interplay between sequence motifs and structural dynamics directly informs classification systems. The classification of LEA proteins is complex due to divergent criteria, primarily based on sequence motifs or polar amino acid composition (Zheng et al., 2019). The widely adopted Battaglia framework categorizes LEA proteins into seven groups based on distinct domain architectures and characteristic motifs (Battaglia et al., 2008), the key features of which are summarized in **Table 1**.

**Table 1:** Classification and characteristic features of LEA protein groups based on the Battaglia framework.

Group	Common name	Key defining features/characteristic motifs	Structural notes
1	–	20-aa motif: TRKEQ[L/M]G[T/E]EGY[Q/K]EMGRKGG[L/E]	–
2	Dehydrins (DHNs)	Lysine-rich 15-aa K-segment: EKKGIMDKIKEKLPG	Predicted to form α-helical structures
3	–	11-mer hydrophobic motif: FF[E/Q]XFK[E/Q]KFX[E/D/Q]^1^	–
4	–	N-terminal α-helix-forming domain; disordered C-terminal region	–
5	–	Lacks distinctive conserved motifs	–
6	–	Conserved domain 1: LEDYKMQGYGTQGHQQPKPGRG Conserved domain 2: GSTDAPTLSGGAV	Low molecular weight
7	ASR proteins	ABA-water deficit stress (ABA/WDS) domain	Absent in *Arabidopsis thaliana*

¹X denotes any amino acid; F represents hydrophobic residues.

Although useful, the Battaglia framework faces challenges when applied across diverse plant species. Extensive studies have revealed systematic discrepancies between its theoretical groups and empirically defined subfamilies. For example, 51 *Arabidopsis thaliana LEA* genes were classified into nine subfamilies, with two unclassified proteins assigned to the AtM subgroup (Hundertmark and Hincha, 2008). Additionally, 29 *Solanum tuberosum LEA* genes were categorized into nine subfamilies (Charfeddine et al., 2015), and 61 *Salvia miltiorrhiza LEA* genes were classified into seven subfamilies (Chen et al., 2021). For a comprehensive comparison across species, please refer to **Table 2**. To address species-specific variations while maintaining a domain-based classification, specialized resources such as the LEAPdb database (Hunault and Jaspard, 2010) have been developed. LEAPdb aids in the organization of hydrophilin data, classification of LEA proteins, functional experimentation, and structure-function analysis (Hunault and Jaspard, 2010).

**Table 2:** LEA proteins in different plants.

Species	Members of the LEA proteins	Number of subfamily (group)	References
*Arabidopsis thaliana*	51	9	(Hundertmark and Hincha, 2008)
*Solanum tuberosum*	29	9	(Charfeddine et al., 2015)
*Salvia miltiorrhiza*	61	7	(Chen et al., 2021)
*Oryza sativa*	34	7	(Wang et al., 2007)
*Citrillus lanatus*	73	4	(Celik Altunoglu et al., 2017)
*Cucumis melo*	61	3	(Celik Altunoglu et al., 2017)
*Camellia sinensis*	33	7	(Wang et al., 2019a)
*Triticum aestivum*	281	8	(Zan et al., 2020)
*Secale cereale*	112	8	(Ding et al., 2021)
*Phyllostachys edulis*	23	6	(Huang et al., 2016)
*Sorghum bicolor*	68	8	(Nagaraju et al., 2019)
*Citrus sinensis*	72	7	(Pedrosa et al., 2015)
*Solanum lycopersicum*	60	8	(Jia et al., 2022)
*Brassica napus*	306	8	(Wang et al., 2024a)
*Dendrobium officinale*	17	7	(Ling et al., 2016)
*Cucumis sativus*	79	7	(Celik Altunoglu et al., 2016)
*P. armeniaca L. × P. sibirica L.* *Malus domestica*	54 87	8 7	(Li et al., 2024) (Wang et al., 2024b)

In conclusion, the defining features of LEA proteins include hydrophilicity, intrinsic disorder, and stress-responsive conformational shifts, such as dehydration-induced α-helix folding. These characteristics form the molecular basis of their role in plant stress adaptation. Moreover, evolved classification systems, which integrate domain-based frameworks with cross-species databases like LEAPdb, facilitate the systematic decoding of structure-function relationships. This integration accelerates research on stress resistance mechanisms.

## 3 Spatiotemporal expression and functions of LEA proteins under stress conditions

**Figure 1:**
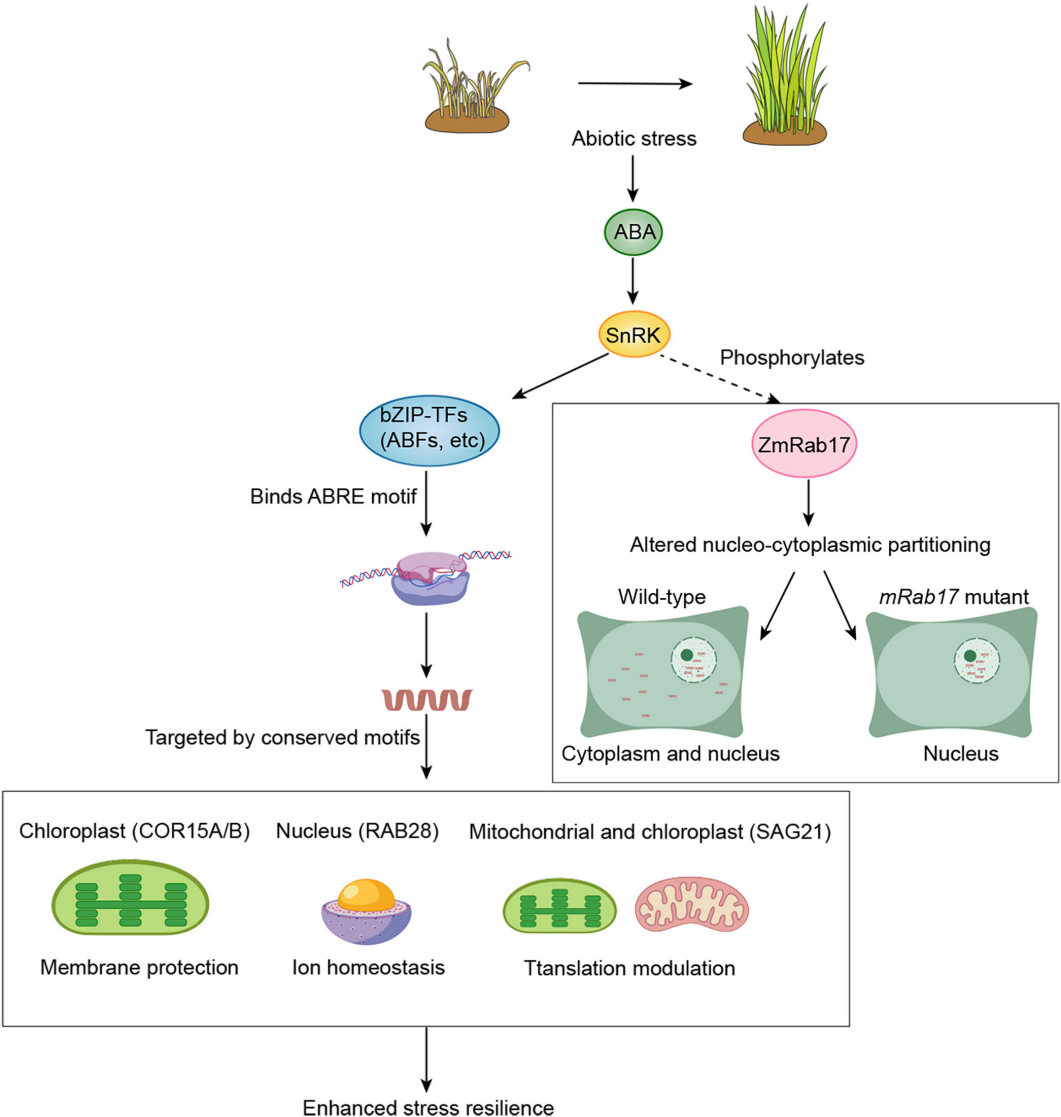
Spatiotemporal expression, regulation, and compartmentalized functions of LEA proteins under abiotic stress. Abiotic stress triggers the accumulation of ABA, which activates SnRK2 kinases, leading to the phosphorylation of ABF transcription factors. These activated ABFs bind to ABRE motifs, thereby enhancing the expression of LEA proteins. LEA proteins are directed to specific subcellular compartments through structurally conserved targeting motifs, facilitating organelle-specific protection. Furthermore, the phosphorylation status of LEA proteins plays a critical role in their localization. For instance, in the case of ZmRab17, the wild-type protein localizes to both the cytoplasm and nucleus, while a phospho-deficient mutant (*mRab17*) accumulates in the nucleolus, illustrating the dynamic regulation of LEA protein subcellular distribution. Arrows denote positive regulation, where solid lines depict well-defined pathways and dashed lines represent speculative relationships. This figure was created using BioGDP.

Structurally conserved motifs, which define LEA protein classification, govern their subcellular localization, enabling compartmentalized functions. Studies indicate that LEA proteins are distributed across various subcellular compartments (Candat et al., 2014; Ginsawaeng et al., 2021). This compartmentalized distribution of LEA proteins enables their direct involvement in protecting critical cellular components within specific organelles (Candat et al., 2014). They respond to stress signals, including those initiated by the key stress hormone ABA (**Figure 1**). Thirty-six Arabidopsis LEA proteins localize to the cytoplasm, and the majority are capable of nucleocytoplasmic trafficking into the nucleus (Candat et al., 2014). This dual positioning places them at the critical interface between cytoplasmic ABA signaling and ABA-triggered nuclear transcriptional reprogramming, suggesting potential direct regulation by ABA or their roles as downstream effectors. Phosphorylation plays a dynamic role in regulating LEA protein localization, as exemplified by maize Rab17. The wild-type protein localizes to the cytoplasm and nucleus, while the non-phosphorylatable mutant (*mRab17*) accumulates in the nucleolus (Riera et al., 2004). Since SNF1-related protein kinase 2 (SnRK2) are central to ABA signaling, ABA likely affects LEA protein localization and function through SnRK2-mediated phosphorylation.

LEA proteins are localized to specific subcellular regions, forming protective zones. Their ABA-regulated expression ensures precise timing, which enables rapid defense mobilization at stress sites. Genome-wide profiling of Arabidopsis *LEA* genes reveals two key patterns related to ABA-driven transcriptional control: first, organ-specific expression, with the highest levels in seeds, reflecting ABA’s role in dormancy; second, ABA/drought inducibility, as the promoters of most *LEA* genes contain ABRE motifs, which trigger rapid upregulation (Zheng et al., 2019; Hu et al., 2024). A strong correlation has also been observed between LEA protein accumulation and plant water deficit, further emphasizing their functional importance under water-limited conditions (Olvera-Carrillo et al., 2010; Guo et al., 2023). The expression patterns and structural features of LEA proteins suggest that they protect plant cells during dehydration and other stress conditions (Hunault and Jaspard, 2010; Olvera-Carrillo et al., 2010). This ABA-mediated spatiotemporal regulation supports LEA proteins as key molecular effectors in stress resilience.

Exploring the functions of these proteins helps deepen our understanding of plant adaptation to stress. *Arabidopsis thaliana* is a key model for studying the functions of LEA proteins, as shown in **Table 3** (Kovacs et al., 2008; Thalhammer et al., 2010). Studies have shown that *LEA13* and *LEA30* enhance water stress tolerance by modulating stomatal density (López-Cordova et al., 2021). *LEA4-2/LEA18* plays a key role in membrane stability (Hundertmark et al., 2011). *COLD-REGULATED 15A* (*COR15A*) and *COR15B* stabilize chloroplast membranes under freezing stress, protecting cells from cold-induced damage (Thalhammer et al., 2010; Navarro-Retamal et al., 2018; Hernández-Sánchez et al., 2024). *RESPONSIVE TO ABSCISIC ACID 28 (RAB28)* is crucial for ion homeostasis during late embryogenesis and germination, highlighting its role in early development (Borrell et al., 2002). *LOW-TEMPERATURE-INDUCED 30* (*LTI30*) protects cellular membranes from dehydration-induced damage (Gupta et al., 2019), while *SENESCENCE-ASSOCIATED GENE 21* (*SAG21*) enhances stress tolerance by modulating mitochondrial and chloroplast translation, underscoring its role in resilience (Karpinska et al., 2022).

**Table 3:** Functions of LEA proteins.

Species	Names	Function	Mechanism	References
*Arabidopsis thaliana*	*LEA13, LEA30*	Enhance water stress tolerance	Modulate stomatal density	(López-Cordova et al., 2021)
*Arabidopsis thaliana*	*LEA4-2/LEA18*	Modulate membrane stability	Anionic membrane-induced β-sheet folding and destabilization	(Hundertmark et al., 2011)
*Arabidopsis thaliana*	*COR15A*, *COR15B*	Freezing protection	Chloroplast membrane stabilization	(Thalhammer et al., 2010; Navarro-Retamal et al., 2018; Hernández-Sánchez et al., 2024)
*Arabidopsis thaliana*	*RAB28*	Maintain ion homeostasis	Regulate cation balance	(Borrell et al., 2002)
*Arabidopsis thaliana*	*LTI30*	Prevent dehydration damage	Membrane protection	(Gupta et al., 2019)
*Arabidopsis thaliana*	*SAG21*	Enhance growth stress tolerance	Modulate organellar translation	(Karpinska et al., 2022)
*Oryza sativa*	*OsLEA3-2*	Enhance drought tolerance	–	(Duan and Cai, 2012)
*Triticum aestivum*	*WZY3-1*	Enhance drought tolerance	–	(Yu et al., 2019)
*Phellodendron amurense*	*TaLEA3*	Improve drought resistance	Regulate stomatal closure	(Yang et al., 2018)
*Capsicum annuum*	*CaDIL1*	Reduce drought tolerance	Impair ABA sensitivity	(Lim et al., 2018)
*Glycine max*	*GmLEA4_19*	Increase drought tolerance	–	(Guo et al., 2023)
*Zea mays*	*ZmDHN15*	Enhance cold tolerance	Reduce oxidative damage and electrolyte leakage	(Chen et al., 2022)
*Oryza sativa*	*OsLEA1a*	Protect membranes	Strengthen antioxidant defenses	(Wang et al., 2021)
*Zea mays*	*ZmDHN1*	Stabilize cellular components	Phospholipid binding with α-helical increase	(Koag et al., 2009)
*Ammopiptanthus mongolicus*	*AmDHN4*	Enhance multi-stress tolerance	–	(Liu et al., 2024)
*Arabidopsis thaliana*	*AtLEA3-3*	Improve salt/osmotic tolerance	–	(Zhao et al., 2011)
*Arabidopsis thaliana*	*LEA4-5*	Reduce osmotic tolerance	Negatively regulated by BPC2	(Li et al., 2021)

These detailed mechanistic insights into the function of LEA proteins in Arabidopsis provide a crucial foundation for understanding their broader significance. Building on this knowledge, research has increasingly focused on exploring the potential of manipulating *LEA* gene expression to enhance stress tolerance, particularly drought resistance, in various plant species. Transgenic overexpression of *OsLEA3–2* in *Oryza sativa* and the heterologous expression of wheat *WZY3–1* in *Arabidopsis thaliana* enhance drought tolerance (Duan and Cai, 2012; Yu et al., 2019). Functional characterization shows that *TaLEA3* enhances drought resistance in *Phellodendron amurense* by promoting faster stomatal closure (Yang et al., 2018). In contrast, reduced expression of *Capsicum annuum Drought INDUCED LATE EMBRYOGENESIS ABUNDANT PROTEIN 1* (*CaDIL1*) in pepper weakens drought tolerance and ABA sensitivity (Lim et al., 2018). Guo et al. found that *GmLEA4_19* overexpression enhances drought tolerance in both Arabidopsis and soybean (Guo et al., 2023).

A wealth of functional evidence underscores the critical contribution of LEA proteins to plant survival under low-temperature stress. For instance, overexpression of *ZmDHN15* in Arabidopsis enhances low-temperature tolerance (Chen et al., 2022). This is demonstrated by reduced malondialdehyde content, lower relative electrolyte leakage, decreased reactive oxygen species (ROS) accumulation, and improved seed germination and seedling survival rates compared to wild-type plants. Additionally, the stress-responsive gene *OsLEA1a* protects cellular membranes and strengthens antioxidant defenses under stress conditions (Wang et al., 2021). Maize DHN1 interacts with anionic phospholipid vesicles. This interaction is associated with an increase in the protein’s α-helical content (Koag et al., 2009). This conformational change is believed to contribute to membrane stabilization and the protection of other cellular components during stress. Similarly, *AmDHN4* overexpression enhances tolerance to low temperature, drought, and osmotic stress in Arabidopsis (Liu et al., 2024).

In addition to their direct protective roles, some LEA proteins also modulate stress signaling pathways. For instance, overexpressing *AtLEA3–3* in Arabidopsis enhances tolerance to salt and osmotic stress, while also increasing sensitivity to ABA (Zhao et al., 2011). Moreover, the regulation of *LEA* gene expression itself plays a key role in stress tolerance. Specifically, the transcription factor BASIC PENTACYSTEINE2 (BPC2) reduces osmotic stress tolerance in Arabidopsis by repressing the expression of *LEA4-5* (Li et al., 2021). This example highlights the complexity of the regulatory networks controlling LEA-mediated stress responses.

In summary, LEA proteins serve diverse functions in plant stress responses. Experimental evidence demonstrates that overexpressing LEA proteins enhances tolerance to drought, freezing, salt, and osmotic stress in transgenic plants, further highlighting their essential role in plant stress resistance (Hu and Xiong, 2014). LEA proteins are known to protect plants from abiotic stresses through multiple mechanisms, including acting as molecular chaperones, stabilizing membranes, and regulating ion homeostasis (Szlachtowska and Rurek, 2023; Hsiao, 2024). However, accumulating evidence indicates that LEA proteins also function as regulatory components within ABA signaling pathways, playing a critical role in mediating abiotic stress responses. Their functional importance is closely tied to their involvement in ABA signaling, which coordinates adaptive responses to environmental challenges. In the following section, we will examine the regulatory relationship between LEA proteins and ABA in detail.

## 4 Regulatory relationship between LEA proteins and ABA signaling

### 4.1 Regulation of *LEA* expression by ABA

**Figure 2:**
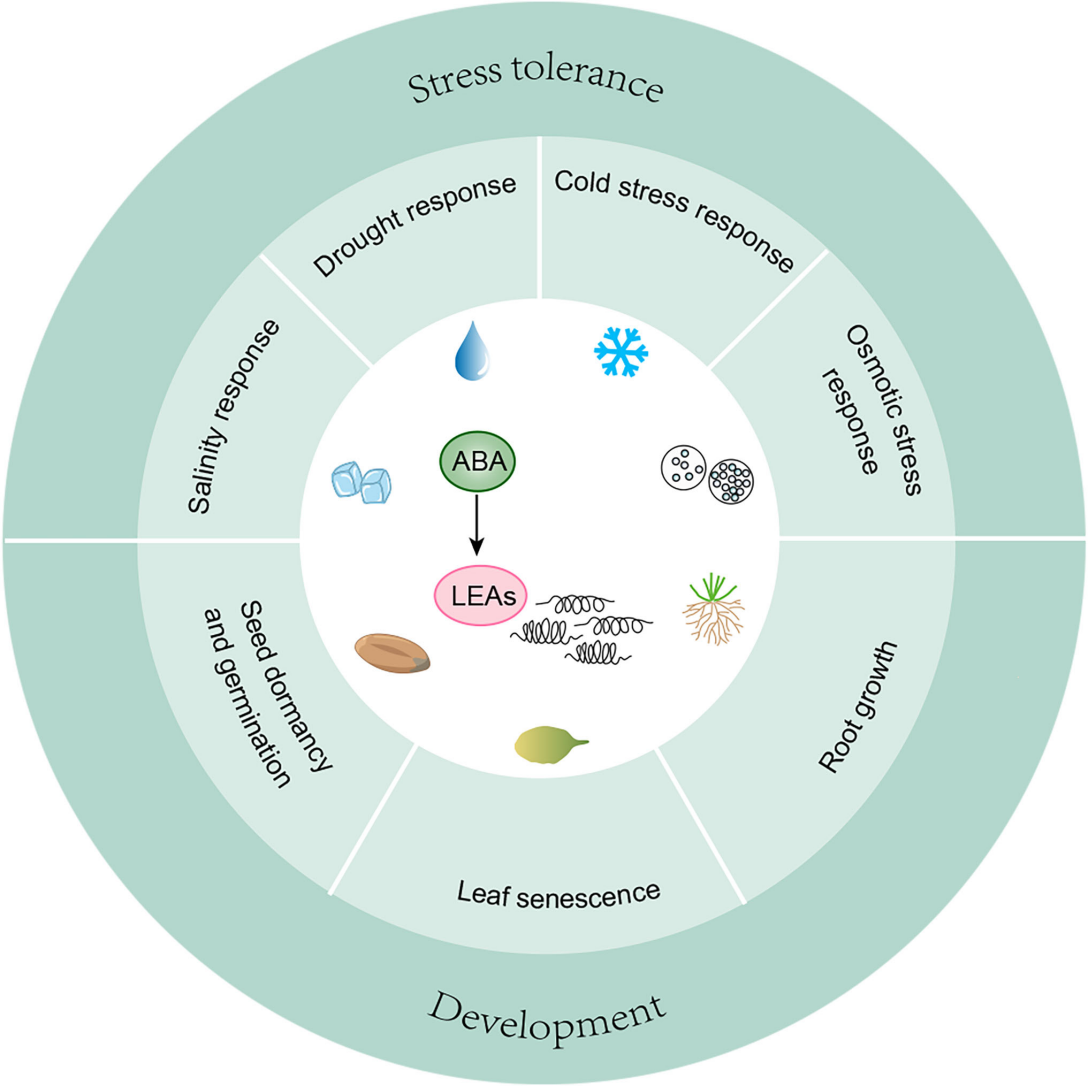
ABA-mediated regulation of LEA proteins in plant stress adaptation and developmental processes. The schematic illustrates the coordinated mechanisms through which plants respond to environmental stresses and developmental cues via ABA-mediated LEA protein expression. It highlights key functional roles of LEA proteins in stress adaptation, including responses to drought, salinity, cold, and osmotic stress, as well as their involvement in various developmental processes such as seed dormancy, germination, root growth, and leaf senescence. Arrows denote positive regulation, where solid lines depict well-defined pathways. The seed elements were created using BioGDP.

The transcription of *LEA* genes is significantly induced by ABA (**Table 4**). As a key component of the ABA signaling pathway, the promoter regions of most *LEA* genes contain abscisic acid response elements (ABREs), which are recognized by ABRE binding factors/ABRE-binding proteins (ABFs/AREBs) (Liu et al., 2019b; Huang et al., 2022). For example, the transcription factor ABA INSENSITIVE 5 (ABI5) binds to ABREs in the promoters of LATE EMBRYOGENESIS ABUNDANT1 (*EM1/LEA1*) and *EM6/LEA6* during seed germination. The application of exogenous ABA enhances the binding affinity of ABI5 to the *EM6* promoter (Carles et al., 2002; Chen et al., 2012). Furthermore, the rice dehydrin *OsDhn-Rab16D*, whose promoter contains multiple ABREs, is inducible by ABA. OsDhn-Rab16D interacts with rice FK506 BINDING PROTEIN (OsFKBP), a prolyl cis-trans isomerase. This interaction, mediated by the ABA signaling pathway, enhances drought tolerance in rice (Tiwari et al., 2019). A model summarizing the ABA-mediated regulation of LEA proteins and their functional roles is presented in **Figure 2**.

**Table 4:** Function and mechanism of LEA proteins regulated by ABA.

Species	Names	Function	Mechanism	References
*Arabidopsis thaliana*	*EM1*	Seed germination	ABA signaling	(Carles et al., 2002)
*Arabidopsis thaliana*	*EM6*	Seed germination	ABA signaling	(Carles et al., 2002; Chen et al., 2012)
*Oryza sativa*	*RAB16D*	Drought tolerance	ABA signaling	(Tiwari et al., 2019)
*Oryza sativa*	*RAB21*	Water stress	ABA signaling	(Mundy and Chua, 1988)
*Arabidopsis thaliana*	*LEA5*	Oxidative stress tolerance	ABA synthesis	(Mowla et al., 2006)
*Arabidopsis thaliana*	*RAB18*	Freezing tolerance	ABA-Dependent	(Lång and Palva, 1992; Mantyla et al., 1995; Nylander et al., 2001)
*Arabidopsis thaliana*	*ABR*	Leaf senescence	ABA signaling	(Su et al., 2016)
*Triticum aestivum*	*SMP1*	Seed dormancy and germination	ABA signaling	(Xu et al., 2025)
*Oryza sativa*	*RePRPs*	Root growth	ABA signaling	(Tseng et al., 2013; Hsiao et al., 2020)
*Medicago Sativa*	*LEA-D34*	Abiotic stress responses and flowering time	ABA signaling	(Lv et al., 2021)
*Medicago falcata*	*LEA3*	Cold and drought tolerance	ABA synthesis	(Shi et al., 2020)
*Vitis vinifera*	*DHN1/DHN2*	Cold-hardiness in dormant buds	ABA and low temperatures	(Rubio et al., 2019)

In Arabidopsis mutants deficient in ABA biosynthesis or signaling, the expression of *LEA* genes has been consistently down-regulated. Proteomic analysis showed a reduction in the expression levels of six out of eight LEA proteins in the embryos of the ABA-deficient mutant *viviparous-5* (*vp5*) (Wu et al., 2014). The promoter activity of *RAB17* is reduced in the ABA-deficient mutant *aba1* compared to wild-type plants and ABA-insensitive mutants (Vilardell et al., 1994). Treatment with ABA or NaCl significantly induce *RAB21* expression in rice (Mundy and Chua, 1988). Drought-induced expression of *AtLEA5* requires ABA synthesis but is independent of ABI1 (Mowla et al., 2006). In *Arabidopsis thaliana*, exogenous ABA promotes *RESPONSIVE TO ABA 18* (*RAB18*) mRNA accumulation (Lång and Palva, 1992). However, *RAB18* expression is delayed in the ABA-insensitive mutant *abi1* and completely absent in *aba1* (Mantyla et al., 1995). Notably, *RAB18* levels show no difference from the wild type in *abi3* mutants, suggesting that *RAB18* expression is ABA-dependent but independent of ABI3 (Nylander et al., 2001).

The expression of LEA proteins is regulated by the core ABA signaling pathway. In Arabidopsis lines overexpressing *CsSnRK2.5* from tea plant (*Camellia sinensis*), ABA treatment and drought stress significantly elevated expression of stress-responsive genes (*AtRAB18*, *AtRD29B*) compared to wild-type plants (Zhang et al., 2020b). Similarly, Arabidopsis overexpressing grape *ABSCISIC ACID RESPONSE ELEMENT-BINDING FACTOR2* (*VvABF2*) from *Vitis vinifera* showed upregulated expression of *RAB18*, *DEHYDRIN LEA* (*LEA*) and *RESPONSIVE TO DESICCATION 29B* (*RD29B*) following ABA treatment (Liu et al., 2019d). Conversely, the *areb1 areb2 abf3* triple mutant exhibits downregulation of *LEA* genes (*RD29B*, *RAB18*, *EM1*, *EM6*) under dehydration, high salinity, or ABA treatment (Yoshida et al., 2010). Drought stress upregulated *RESPONSIVE TO DESICCATION 29A* (*RD29A*), *RD29B*, *COLD-REGULATED 47* (*COR47*), *RAB18*, and RESPONSIVE TO DESICCATION 22 (*RD22*) in *IbABF4*-overexpressing Arabidopsis and sweet potato (*Ipomoea batatas*) (Wang et al., 2019b). MYB DOMAIN PROTEIN 44 (MYB44) interacts with REGULATORY COMPONENT OF ABA RECEPTOR 1/PYRABACTIN RESISTANCE 1-LIKE 9 (RCAR1/PYL9) to attenuate ABI1 phosphatase inhibition, thereby negatively regulating *RAB18* expression (Li et al., 2014a). Under salt stress, *GhMYB73*-overexpressing Arabidopsis shows elevated *RD29B* transcription. This effect may involve GhMYB73-PYL8 interaction modulating *RD29B* expression (Zhao et al., 2019). Arabidopsis LEA family members, including *ABA-RESPONSIVE PROTEIN* (*ABR*), are strongly induced by ABA, NaCl, and mannitol. ABR serves as a marker for ABA signaling and participates in ABI5-mediated leaf senescence (Tanaka et al., 2012; Su et al., 2016). Dehydrins contain SnRK2-specific phosphorylation sites. Notably, the ABA-nonactivated kinase SnRK2.10 phosphorylates Early Responsive to Dehydration 10 (ERD10) and ERD14 under osmotic stress (Maszkowska et al., 2019).

Emerging evidence indicates that multiple LEA proteins participate in abiotic stress responses through specific protein interactions. For example: in wheat, the dehydrin WZY2 (GenBank NO. EU395844) promoter contains ABRE, and WZY2 interacts with a PP2C phosphatase (XM_020293398). These features suggest WZY2 regulates abiotic stress-responsive genes via the ABA pathway (Zhu et al., 2014; Liu et al., 2019c). As a LEA family member, TaSMP1 interacts with ABI5 to modulate expression of the seed germination gene *DOG1L1*, thereby regulating seed dormancy and germination (Xu et al., 2025). In rice, the ABA-induced REPETITIVE PROLINE-RICH PROTEIN (RePRP) interacts with the cytoskeleton to facilitate adaptive root growth under stress conditions (Tseng et al., 2013; Hsiao et al., 2020). Furthermore, ABA signaling acts as a central hub for indirectly modulating LEA protein accumulation. ELONGATED HYPOCOTYL 5 (HY5), a pivotal transcription factor in light signaling, promotes *LEA* genes expression by directly binding to the *ABI5* promoter. This integration of light and ABA signaling enhances seedling tolerance to drought, salinity, and low temperature (Chen et al., 2008). *DELAY OF GERMINATION 1* (*DOG1*), a key regulator of seed dormancy, induces *LEA* genes expression during seed development through ABI5-mediated regulation (Dekkers et al., 2016).

### 4.2 Multiple signaling pathways regulate LEA Proteins through ABA-mediated cross-talk

The expression of *LEA* genes is coordinately regulated by a sophisticated network, where ABA signaling serves as a central hub integrating diverse environmental and intracellular cues. Environmental signals, such as low temperature, initiate this regulatory network through synergistic interplay with ABA. Exogenous ABA application induces the expression of multiple cold stress-responsive dehydrin genes in *Arabidopsis thaliana*, with differential regulatory effects on distinct dehydrin subtypes (Guo et al., 1992; Rouse et al., 1996; Wang et al., 2014). This synergy is evident as ABA synthesis inhibitors block the low temperature induction of *MfLEA3* (Shi et al., 2020), and combined ABA-cold treatment regulates the expression of *VvDHN1* and *VvDHN2* to enhance cold hardiness in grapevine (Rubio et al., 2019). This cross-talk is often mediated by key transcription factors. For instance, MsABF2 directly binds to the promoter of *MsLEA-D34* to activate its expression (Lv et al., 2021), while DREB/CBF-type factors like VaCBF4 and OsDREB1F integrate ABA and stress signals, either directly or indirectly, to activate canonical ABA-responsive *LEA* genes such as *RD29A*, *COR47*, and *RAB18* (Li et al., 2013; Wang et al., 2008).

Beyond environmental perception, intracellular second messengers, particularly calcium (Ca^2+^), form a critical layer of regulation. Stress-induced Ca^2+^ fluctuations are decoded by sensor proteins including Ca^2+^-dependent protein kinases (CPKs/CDPKs), calcineurin B-like protein complexes (CBL-CIPK), calmodulin-like proteins (CMLs), and calmodulins (CaMs) (Kudla et al., 2018), which subsequently regulate gene expression via MAPK cascades or transcription factors (Sun et al., 2021).

The CPK/CDPK branch acts as a central integrator, primarily by phosphorylating ABA signaling components. Arabidopsis CPK32 phosphorylates ABF4 to activate *RD29A/RAB18* expression (Choi et al., 2005), while CPK4/11 target ABF1/ABF4 (Zhu et al., 2007), with *cpk1* mutants showing impaired *RD29A*/*COR15A* expression (Huang et al., 2018a). The wheat TaCDPK9 module regulates ABA biosynthesis (Zhang et al., 2020a), establishing a feedback loop where CPK-phosphorylated ABFs drive *LEA* expression while LEA proteins modulate Ca^2+^ signaling through ABA homeostasis (Liu et al., 2022).

The CBL-CIPK module provides additional integration points. TaCIPK27 upregulates *RD29B* and other ABA-responsive genes (Wang et al., 2018), while CIPK3 mediates ABA-cold crosstalk for *RD29B/RD29A* induction and interacts with ABR1 to link Ca^2+^ and ABA signaling (Kim et al., 2003; Sanyal et al., 2017). CML20 functions as a negative regulator, with its mutation upregulating *RAB18*/*COR47* expression (Wu et al., 2017).

MAPK cascades also regulate *LEA* genes, as demonstrated by reduced *COR15A*/*RD29A* in cold-stressed *mpk3*/*mpk6* mutants (Li et al., 2017) and impaired *RD29B*/*RAB18* induction in ABA-treated *mkkk18* mutants (Mitula et al., 2015).

In conclusion, *LEA* expression is fine-tuned by a multi-layered regulatory network. This network seamlessly integrates direct environmental signals with intracellular second messengers (Ca^2+^) and kinase cascades (MAPK), with the ABA signaling pathway acting as the central backbone for this extensive cross-talk, ensuring a robust and adaptable stress response.

### 4.3 Feedback regulation of the ABA signaling by LEA proteins

Recent studies have revealed that LEA proteins are not merely passive effectors of ABA signaling but actively regulate the ABA pathway through feedback mechanisms (**Table 5**). Multiple LEA proteins (*CaLEA1*, *LsEm1*, and *AtruLEA1*) regulate stress responses through ABA sensitivity (Lim et al., 2015; Xiang et al., 2018; Li et al., 2025). These proteins participate in fine-tuning ABA accumulation and homeostasis. For example, Overexpression of the dehydrin *TAS14* increases ABA accumulation in leaves during short-term stress (Muñoz-Mayor et al., 2012). LEA12^OR^ stabilizes the STRESS/ABA-ACTIVATED PROTEIN KINASE (SAPK10) under salt stress, promoting ABA biosynthesis and enhancing salt tolerance in rice (Ge et al., 2024). The LEA-like protein Salt Tolerance–Related Protein (STRP) regulates ABA sensitivity. The *strp* mutants exhibit defects in ABA responses, including germination, root growth, and stomatal closure, and show reduced expression of *NINE-CIS-EPOXYCAROTENOID DIOXYGENASE 3* (*NCED3*) under salt stress (Fiorillo et al., 2020, 2023). OsLEA5 enhances drought tolerance by promoting ABA accumulation through upregulating ABA biosynthesis genes (*NCED1*, *NCED5*) and inhibiting ABA catabolism genes (*ABA8ox2*). It also interacts with ZINC FINGER PROTEIN 36 (ZFP36) to activate ABA-mediated antioxidant defense, improving drought and salt stress adaptation, and contributes to ABA-dependent seed germination inhibition (Huang et al., 2018b, 2018c).

**Table 5:** Function and mechanism of feedback regulation of ABA by LEA proteins.

Species	Names	Functions	Mechanism	References
*Capsicum annuum*	*LEA1*	Drought and salt stress	ABA signaling	(Lim et al., 2015)
*Lactuca sativa*	*Em1*	Drought and salt stress	ABA signaling	(Xiang et al., 2018)
*Acer truncatum*	*LEA1*	Drought and salt tolerance	ABA sensitivity	(Li et al., 2025)
*Solanum lycopersicum*	*TAS14*	Drought and salt stress	ABA accumulation	(Muñoz-Mayor et al., 2012)
*Oryza rufipogon*	*LEA12^OR^*	Salt tolerance and yield	ABA synthesis	(Ge et al., 2024)
*Arabidopsis thaliana*	*STRP*	Salt stress	ABA synthesis	(Fiorillo et al., 2020, 2023)
*Oryza sativa*	*LEA5*	Antioxidant defense	ABA biosynthesis and ABA metabolism	(Huang et al., 2018c)
*Oryza sativa*	*LEA5*	Seed germination	ABA signaling	(Huang et al., 2018b)
*Arabidopsis thaliana*	*LTI30*	drought stress	ABA sensitivity	(Shi et al., 2015)
*Triticum aestivum*	*HVA1*	Drought and heat stress	ABA sensitivity	(Samtani et al., 2022)
*Arabidopsis thaliana*	*LEA14*	Drought stress	ABA signaling	(Li et al., 2014b)

Beyond their roles in ABA feedback regulation, distinct subgroups of LEA proteins extensively participate in plant adaptive responses to drought, salinity, and temperature stresses. They function by modulating ABA sensitivity or mediating the expression of downstream stress-related genes. The following research cases systematically reveal the multidimensional regulatory mechanisms of LEA proteins in ABA signaling transduction. LTI30, an Arabidopsis dehydrin from Group II LEA proteins, exemplifies this regulation. Knockout mutants of *LTI30* show reduced sensitivity to ABA during seed germination, while overexpression lines show increased ABA sensitivity (Shi et al., 2015). Similarly, overexpression of the *OsEm1* gene increases ABA sensitivity and upregulates the expression of other *LEA* genes, including *RAB16A/C*, *RAB21*, and *LEA3* (Yu et al., 2016). In cotton, knockout of *LEA3* (Gh_A08G0694) increases sensitivity to salt and drought stress and downregulates the expression of ABA/stress-related genes (Shiraku et al., 2022). Furthermore, HORDEUM VULGARE ALEURONE 1 (HVA1), a Group 3 LEA protein, enhances both drought resistance and heat tolerance through a dual regulatory network. Transgenic plants overexpressing *HVA1* also display enhanced sensitivity to ABA (Samtani et al., 2022). Another study shows that overexpression of DHN, a member of the LEA protein family, upregulates genes involved in the ABA signaling pathway, such as *RD22* and *RD29B* (Mota et al., 2019). Collectively, these findings establish LEA proteins as key regulators of plant stress resilience. They regulate ABA signaling cascades and modulate downstream stress-responsive gene networks.

**Figure 3:**
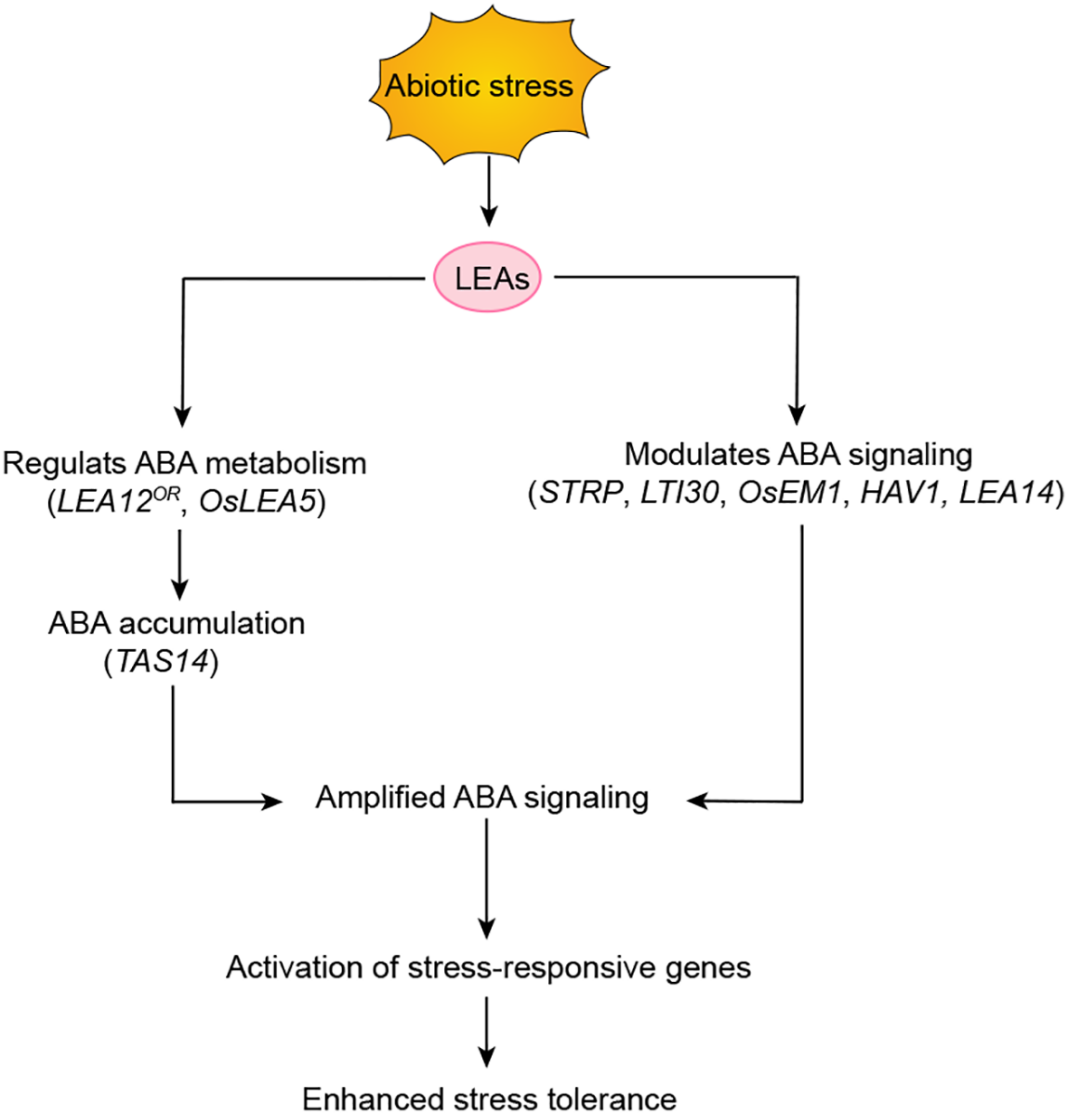
Regulatory roles of LEA proteins in enhancing abiotic stress tolerance through ABA signaling modulation. LEA proteins function as core regulators in plant responses to abiotic stress. They enhance stress tolerance through three primary mechanisms: regulating ABA accumulation (*LEA12^OR^*, *OsLEA5*, *TAS14*), modulating ABA signaling (*STRP*, *LTI30*, *OsEM1*, *HVA1, AtLEA14*). By influencing ABA accumulation and amplifying ABA signaling, LEA proteins promote the activation of stress-responsive genes. These integrated actions collectively enhance plant stress tolerance through coordinated transcriptional reprogramming. Arrows denote positive regulation, where solid lines depict well-defined pathways.

Research demonstrates that LEA proteins indirectly regulate the ABA signaling pathway through protein-protein interaction networks (Dirk et al., 2020). Under drought stress, both *AtLEA14*-overexpressing lines and *atpp2-b11* RNAi lines exhibit enhanced ABA sensitivity. The molecular mechanism likely involves AtLEA14 sequestering the AtPP2-B11 protein. This sequestration indirectly protects SnRK2 kinases from 26S proteasome-mediated degradation, ultimately promoting ABA signaling activation. This interaction reflects a synergistic inhibitory effect between LEA proteins and their partners during drought response (Li et al., 2014b; Cheng et al., 2017). Under salt stress conditions, both overexpression of the AtPP2-B11 F-BOX protein and overexpression of *AtLEA14* significantly improve plant salt tolerance. Further investigations reveal that AtLEA14 maintains the structural stability of the AtPP2-B11 protein in saline environments. The stabilized AtPP2-B11 may then confer stress protection by specifically degrading transcription repressors that negatively regulate salt tolerance (Jia et al., 2014, 2015). Collectively, these findings unveil the molecular mechanism by which LEA proteins achieve environment-specific responses through dynamic protein interaction networks under distinct abiotic stresses, as illustrated in **Figure 3**.

## 5 Conclusions and future perspectives

**Figure 4:**
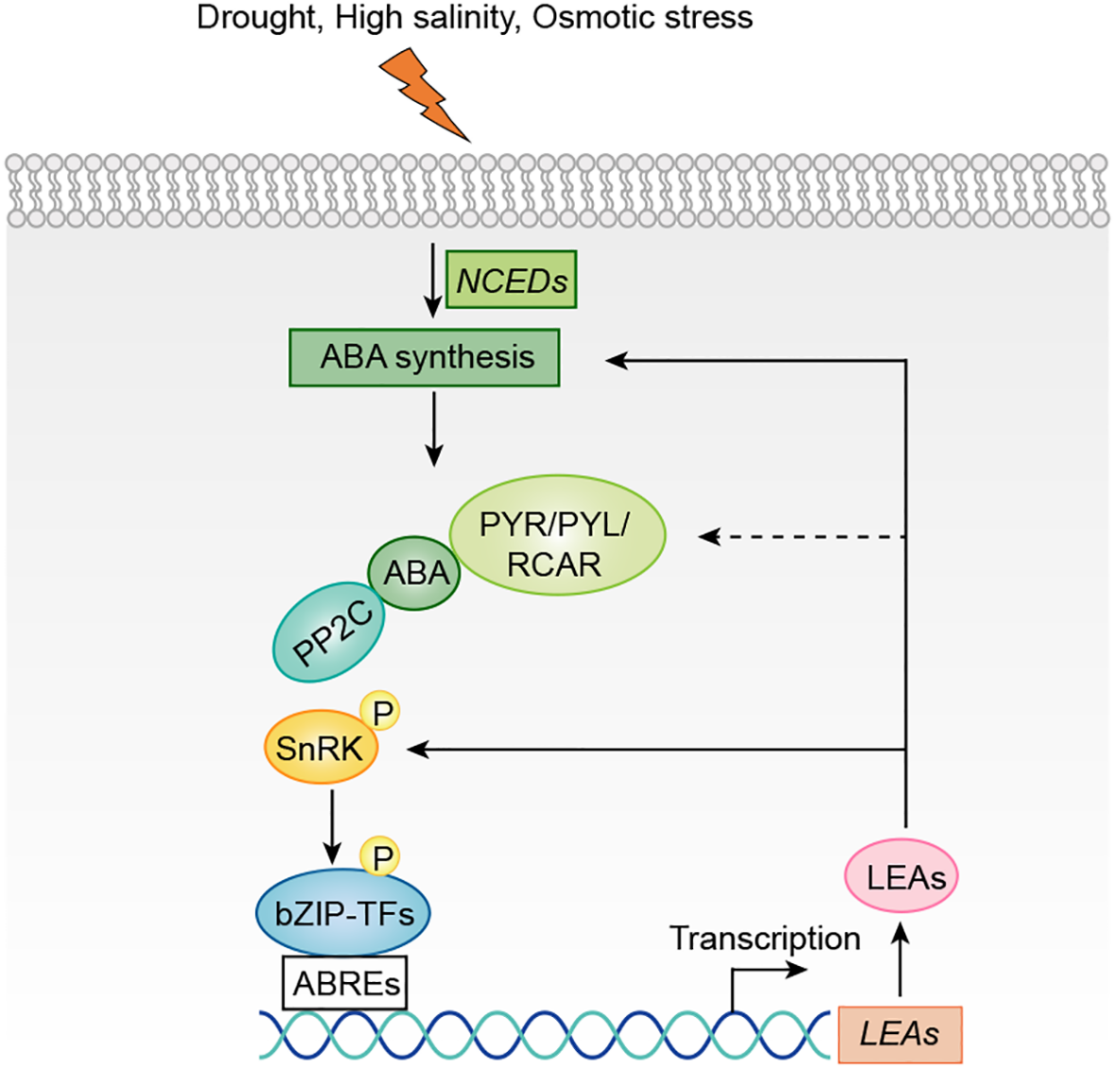
The crosstalk between LEA proteins and ABA under abiotic stress. When plants encounter drought, salinity, or osmotic stress, *NCED* gene expression is upregulated, enhancing ABA biosynthesis. The accumulated ABA is perceived by PYR/PYL/RCAR receptors, which inhibit PP2C phosphatase activity, thereby activating SnRK2 kinases. Activated SnRK2 phosphorylates bZIP transcription factors, enabling their binding to ABRE elements in *LEA* gene promoters and activating *LEA* expression. Subsequently, LEA proteins reinforce ABA signaling by upregulating *NCED* expression and modulating downstream stress-responsive networks, establishing a self-amplifying positive feedback loop that potentiates the plant’s stress adaptation. Arrows denote positive regulation, where solid lines depict well-defined pathways and dashed lines represent speculative relationships.

This review synthesizes multi-source evidence to propose an “ABA-LEA positive feedback loop” model. According to this model, abiotic stresses, including drought, high salinity, and low temperature, activate the ABA signaling pathway and upregulate *LEA* expression. Beyond their conventional protective roles, LEA proteins function as active regulators that physically interact with core ABA signaling components, thereby amplifying the signal output to form a self-reinforcing circuit (**Figure 4**).

**Figure 5:**
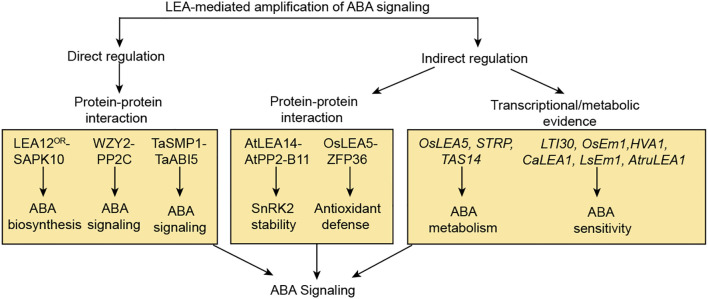
LEA proteins act as active regulators to amplify ABA signaling. This model summarizes the molecular evidence that LEA proteins act as active regulators to amplify ABA signaling. The amplification is achieved via three coordinated strategies: direct protein-protein interactions with core signaling components (LEA12^OR^-SAPK10, WZY2-PP2C, TaSMP1-TaABI5), indirect regulation through intermediary partners (AtLEA14-AtPP2-B11, OsLEA5-ZFP36), and the transcriptional and metabolic regulation of ABA homeostasis and sensitivity by *OsLEA5*, *STRP*, *LTI30* and so on. Collectively, these LEA-driven mechanisms enhance ABA signaling, thereby forming a positive feedback loop that ensures a robust and sustained adaptive response to abiotic stress. Arrows denote positive regulation, where solid lines depict well-defined pathways.

This review proposes an insightful “ABA-LEA positive feedback loop” model integrating traditional views with multi-source evidence. According to this model, abiotic stresses such as drought, high salinity, and low temperature activate the ABA signaling pathway, leading to upregulated *LEA* expression. Beyond their conventional protective roles, LEA proteins also function as active regulators that directly or indirectly interact with core ABA signaling components, thereby amplifying and sustaining ABA signal output and forming a self-reinforcing circuit (**Figure 5**). These include direct physical interactions with core ABA components such as PP2C phosphatases, SnRK2 kinases, and ABI5-like transcription factors, illustrated by WZY2-PP2C fine-tuning of ABA signaling in wheat, LEA12^OR^-SAPK10 stabilization promoting ABA biosynthesis, and TaSMP1-TaABI5 regulation of seed dormancy (Liu et al., 2019c; Ge et al., 2024; Xu et al., 2025). LEA proteins also engage in indirect modulation through interaction partners such as E3 ligases and zinc finger proteins, exemplified by AtLEA14 sequestering AtPP2-B11 to stabilize SnRK2 kinases under drought, OsLEA5 binding ZFP36 to enhance ABA-mediated antioxidant defense, and OsDhn-Rab16D interacting with OsFKBP to improve drought tolerance (Jia et al., 2014; Cheng et al., 2017; Huang et al., 2018b, 2018c; Tiwari et al., 2019). Additionally, several LEA proteins, including *OsLEA5*, *STRP*, *TAS14*, *LTI30*, *OsEm1*, *HVA1*, *CaLEA1*, *LsEm1*, and *AtruLEA1*, modulate ABA sensitivity or accumulation, thereby influencing stress-related phenotypes (Huang et al., 2018c; Fiorillo et al., 2020, 2023; Muñoz-Mayor et al., 2012; Gupta et al., 2019; Yu et al., 2016; Samtani et al., 2022; Lim et al., 2015; Xiang et al., 2018; Li et al., 2025). Collectively, these interactions form a unified “bidirectional ABA-LEA regulatory network” model, wherein LEA proteins reinforce ABA signaling to ensure rapid and robust stress adaptation. By integrating multi-source evidence, this review provides novel insights into the functions and mechanisms of LEA proteins in plants, enhancing our understanding of the molecular basis of plant stress responses and their potential agricultural applications.

The integration of LEA proteins and ABA signaling constitutes a central regulatory network in plant stress adaptation, where LEA members such as *ZmDHN15* and *OsLEA1a* contribute to cellular redox homeostasis alongside their protective functions (Wang et al., 2021; Chen et al., 2022). This LEA-ABA feedback system further interfaces with ROS signaling and epigenetic reprogramming, reinforcing the perspective that ROS act as core elements of the epigenetic regulatory machinery (Kaya and Adamakis, 2025). Within this model, ABA-induced ROS fulfill dual and interconnected roles: they trigger immediate physiological responses such as stomatal closure (Postiglione and Muday, 2020) and drive persistent epigenetic changes, including DNA hypomethylation, which facilitates the activation of stress-responsive genes such as those encoding LEA proteins (Shi et al., 2017). The network is further reinforced as some LEA proteins, exemplified by OsLEA5, enhance ABA signaling and bolster antioxidant defenses (Huang et al., 2018c). Collectively, these interactions establish a “LEA-ABA-ROS-Epigenetic” axis, wherein ROS function as a dynamic hub linking rapid stress transduction to long-term transcriptional tuning via chromatin remodeling, thereby enhancing the plant’s adaptive capacity and stress memory.

Despite the promising potential of this model, its molecular mechanisms and broader biological implications require further systematic investigation. Current research remains largely focused on functionally characterizing *LEA* genes in a limited number of model plants, while a comprehensive understanding of their upstream regulatory networks and functional diversity across species and tissues is still lacking. To advance the field, future studies should prioritize the following three directions.

First, a deeper exploration of the molecular mechanisms governing the ABA-LEA interaction module is essential. Building on known interaction cases, systematic efforts should screen for direct interaction networks between LEA proteins and core ABA components, coupled with structural analyses of these complexes. The regulatory roles of post-translational modifications in LEA function warrant further investigation. For instance, elucidating whether CKII-mediated phosphorylation influences the nuclear localization and function of maize ZmDHN11 (Ju et al., 2021). Research should also examine the potential liquid-liquid phase separation behavior of LEA proteins during stress granule assembly, which could help distinguish their non-canonical regulatory roles from classical chaperone functions (Ginsawaeng et al., 2021; Hernández-Sánchez et al., 2022). Integrating live-cell imaging and single-molecule tracking to visualize the dynamic assembly of these modules *in vivo* will be crucial for confirming their physiological relevance. Furthermore, a critical yet under-explored area is the identification of mechanisms that attenuate or terminate the ABA-LEA positive feedback loop. While our model emphasizes signal amplification, any robust signaling system requires built-in “braking mechanisms” to prevent over-activation and ensure homeostasis. Future research should prioritize uncovering these negative regulatory circuits. Key questions include: How is LEA protein activity itself downregulated post-translationally? Are there specific E3 ubiquitin ligases or proteases that target regulatory LEA proteins for degradation upon stress relief? Does feedback inhibition from other hormone signaling pathways actively suppress the ABA-LEA axis to promote growth recovery? Elucidating these termination signals is not merely an addendum to the model but is fundamental to understanding the dynamic control and plasticity of plant stress responses, completing our holistic view of this regulatory network.

Second, research should expand to examine the evolutionary conservation and functional diversity of the ABA-LEA module. From a comparative and evolutionary perspective, the regulatory module linking ABA signaling to LEA protein expression is deeply conserved across land plants (Shinde et al., 2012). This conservation is observed in both monocots and dicots, where *LEA* gene promoters typically harbor ABA-responsive elements and show ABA-inducible expression (Liu et al., 2019b). Furthermore, key transcription factors such as ABI5 directly activate *LEA* genes, illustrating a shared regulatory logic (Su et al., 2016). Beyond this conserved framework, lineage-specific innovations have subsequently evolved. Monocots have expanded their *LEA* gene families (Zan et al., 2020) and developed novel protein interaction networks (Tiwari et al., 2019), enhancing their stress responsiveness. In contrast, dicots often integrate LEA proteins into broader developmental programs such as leaf senescence and flowering time (Su et al., 2016; Lv et al., 2021), highlighting divergent evolutionary strategies in adapting ABA-LEA signaling to distinct physiological contexts. Building upon this evolutionary foundation, a key future goal is to map the detailed landscape of these adaptations. Integrating cross-species comparative genomics with single-cell multi-omics data will help systematically analyze the conservation, lineage specificity, and tissue-specific expression patterns of this module across diverse plant groups (Battaglia and Covarrubias, 2013; Hernández-Sánchez et al., 2022). It is vital to clarify the functional differentiation of *LEA* subtypes among various cell types, tissues, and key physiological processes such as seed development, dormancy, and germination (Knox-Brown et al., 2020; Zamora-Briseño and de Jiménez, 2016).

Furthermore, elucidating the crosstalk between the ABA-LEA module and other key stress signaling pathways such as calcium signaling and MAPK cascades will be instrumental in constructing a more comprehensive plant stress response network. Calcium signaling acts as an independent second messenger system that engages in multi-level crosstalk with the ABA pathway, cooperatively regulating the expression of *LEA* and other stress-responsive genes. Similarly, core ABA signaling components can activate MAPK cascades, which fine-tune the expression of *LEA* through phosphorylation of ABA-responsive transcription factors (Sun et al., 2021). Although current evidence does not indicate that the ABA-LEA axis can directly feedback-regulate upstream elements such as calcium dynamics or MAPK activity, determining whether LEA proteins possess feedback or signal integration capabilities remains a critical research direction, to be addressed through multi-level approaches spanning protein interactions, transcriptional regulation, and epigenetics.

Third, translating the ABA-LEA module from theoretical concept to agricultural application represents a vital frontier. Building on existing overexpression studies-such as those demonstrating improved drought tolerance conferred by *OsLEA3–1* or *HVA1* (Xiao et al., 2007; Samtani et al., 2022), future work should develop synthetic biology strategies to rationally design *LEA* variants with enhanced interaction capacity or stability. CRISPR-based gene editing could also be employed to precisely modulate key nodes within this regulatory circuit, facilitating the development of novel crop germplasm with enhanced, conditionally regulated stress resilience. As most current studies rely on transgenic overexpression, strengthening reverse genetics validation using *LEA* knockout mutants (López-Cordova et al., 2021; Su et al., 2016) will provide a more robust theoretical foundation for breeding applications.

In summary, redefining LEA proteins as active regulatory components within the ABA represents a significant conceptual advance in plant stress biology. Through interdisciplinary integration of diverse research tools, systematic dissection and rational design of the ABA-LEA module will help bridge the gap from mechanistic insight to practical innovation, offering core technological drivers to address food security challenges under global climate change.
